# Enhancement of photosynthesis in *Synechococcus* bacillaris by sponge-derived Ageladine A

**DOI:** 10.1371/journal.pone.0213771

**Published:** 2019-03-26

**Authors:** Ulf Bickmeyer, Silke Thoms, Florian Koch, Liliane Petety Mukagatare, Romaston Silalahi, Franz Josef Sartoris

**Affiliations:** Alfred Wegener Institute Helmholtz Center for Polar and Marine Research, Department of Biosciences, Bremerhaven, Germany; Helmholtz-Zentrum fur Ozeanforschung Kiel, GERMANY

## Abstract

This study is a proof of concept that the sponge derived pyrrole-imidazole alkaloid Ageladine A acts as an additional light harvesting molecule for photosynthesis of symbionts of marine sponges. The absorbance of Ageladine A is in the UV range and fluoresces blue, matching the blue absorbance of chlorophyll a. A joint modeling and experimental approach demonstrates that Ageladine A increases photosynthetic O_2_ production of *Synechococcus* bacillaris WH5701 (CCMP1333), when the cells are exposed to UV light, which is marginally used for photosynthesis. Due to the presence of Ageladine A, production of O_2_ increased 2.54 and 3.1-fold, in the experiments and the model, respectively.

## Introduction

**Fig 1:**
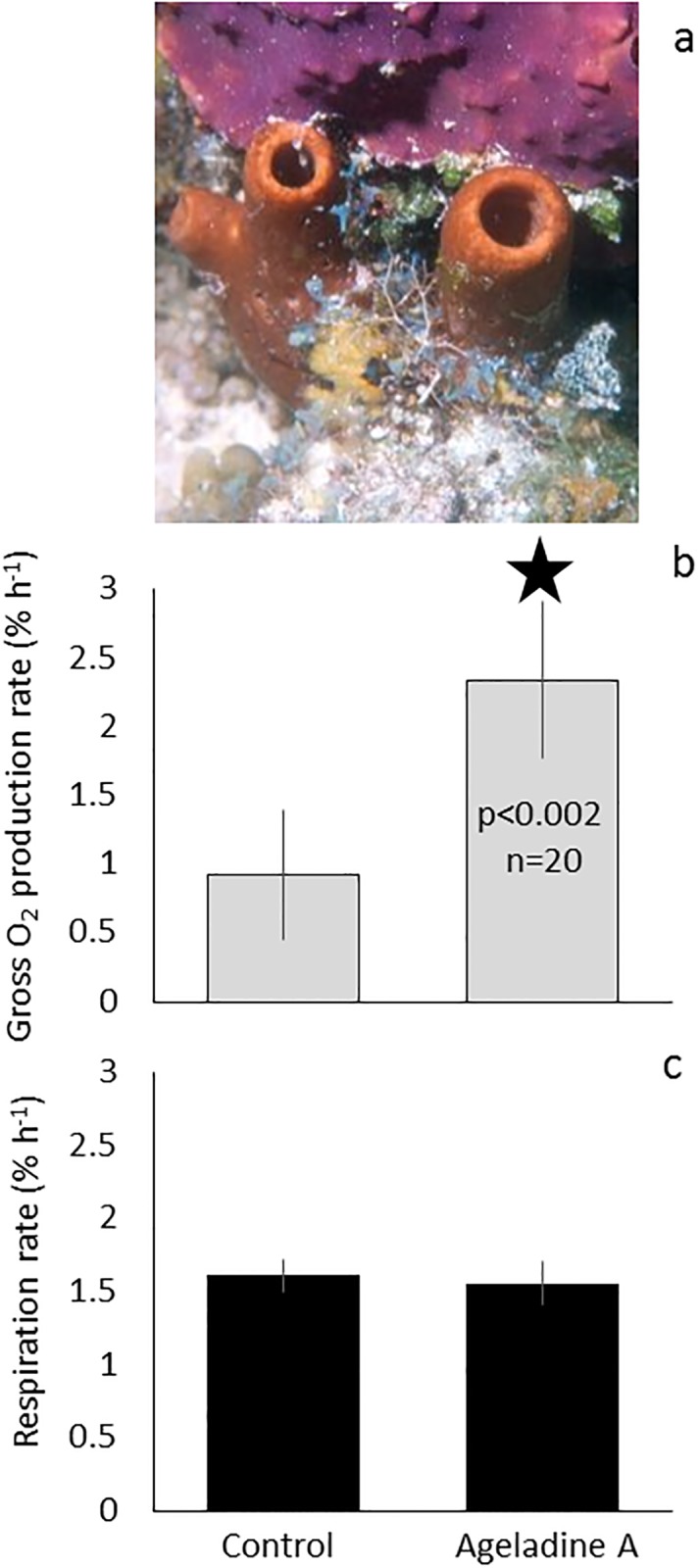
Evidence for a possible type of symbiotic interaction in *Agelas sp*. sponges (**a** courtesy [6]). The addition of 30 μmolL^-1^ Ageladine A resulted in a significant (p<0.002, n = 20) increase of gross O_2_ production rates in cultures of *Synechococcus bacillaris* (**b**). In addition, the respiration rates of these cultures were the same, irrespective of exposure to Ageladine A (**c**). Experiments for the controls and Ageladine A treatments were conducted in parallel and lasted 600 and 90 minutes for the O_2_ respiration and production rate measurements, respectively. Values represent mean ± standard error. The star denotes a significant difference between the treatment and the control.

Marine sponges harbor many symbionts, such as the cyanobacterium *Synechococcus*, which constitute a large amount of the sponge’s dry weight [1,2]. Symbionts of sponges likely play a role in digestion and protection similar to that of the gut microbiome in mammals [1]. Demospongae and associated microbiota produce many different secondary metabolites which serve as feeding deterrents and antibiotics [3]. Many exhibit a specific pharmacological profile [4,5]. The widely distributed sponges of the genus *Agelas* (Fig 1a) [6] produce the fluorescing pyrrole imidazole alkaloid Ageladine A. This compound was first detected and described during the search for an antiangiogenic matrixmetalloproteinases inhibitor [7] and later chemically synthetized [8–11]. Ageladine A [7] accumulates in acidic cellular compartments [12] and has previously been used as a pH sensitive, fluorescent dye [13]. With decreasing pH, Ageladine A is protonated twice, and trapped in acidic cellular compartments, where it fluoresces blue when exposed to UV light [12]. These include lysosomes, endosomes and the thylakoid lumen found in photoautotrophic symbionts, like cyanobacteria [14]. Thus, Ageladine A may also accumulate in the acidic thylakoid space of photosynthetic cells. The ecophysiological role of Ageladine A in the sponge biome is still unresolved. However, its properties suggest that Ageladine A may serves as an antennae molecule, absorbing UV/blue light, transforming it to usable photosynthetic active radiation (PAR), and thereby supporting photosynthesis by the sponges’ symbionts. Results presented here suggest that Ageladine A acts as an additional light harvesting molecule for photosynthesis in *Synchecoccus*. By combining a modeling and experimental approach, it was revealed that Ageladine A facilitates photosynthesis of *Synechococcus*, when the cells are exposed to UV light. This study hypothesizes a type of symbiosis in which light may be the currency of species interaction.

## Materials and methods

### Culture and experimental conditions

The strain WH5701 (CCMP1333) was obtained from Jacky Collier (Stony Brook University, USA) and maintained in F/2 media [15] made from filter sterilized North Sea Seawater (Salinity = 31). Since symbiotic *Synchecoccus* derived from sponges have thus far not been culturable, it is unclear whether they possess similar photosynthetic properties as the strain used in this study. It is assumed, however, that the basic photophysiological properties are constrained amongst various strains.

Cultures were maintained at 20° C and illuminated with 30 μmol photons m^-2^ s^-1^ on a 16:8 light/dark cycle. To ensure an axenic strain, cultures were treated with cycloserine according to [16]. Briefly: cultures were grown in the dark for 48 h after which cycloserine (Sigma) was added at a 1 mg mL^-1^. After an additional 48 h. in the dark, the cultures were gently centrifuged (2000 x g) for 15 minutes, the supernatant discarded and the cell pellet was resuspended with fresh F/2 media. This step was repeated two more times, ensuring that all of the cycloserine was diluted out of the culture media after which cultures were once again returned to the above described conditions. The axenic nature of the culture was sporadically tested by plating cultures on agar solidified media containing 0.5% glucose and 0.05% tryptone to detect heterotrophic contaminants [17]. Cell growth was monitored using a Beckman Coulter Multisizer 3 Coulter Counter with a 30 μm aperture [18]. Subsamples for the tests with Ageladine A were taken at various stages of cell growth (0.3–1.5 x 10^7^ cells mL^-1^) and oxygen consumption/production (expressed as the difference to 100% saturation in air) of *Synechococcus* was measured using optical sensors Microx Tx3 Presens, Regensburg, Germany). Sensors were regularly calibrated using Na_2_SO_3_ (0% O_2_) and saturated seawater (100% O_2_). The oxygen consumption was in the range of fmol per cell per hour. Because our focus was on the relative differences between control and Ageladine A treated samples, we decided to use percent change of oxygen production. Respiration chambers were created by connecting optodes to airtight 3ml quartz cuvettes. The intensity of the excitation radiation was confirmed using a Ramses ARC system (Trios, Rastede, Germany). During preliminary experiments the light intensity and wavelength were adjusted to 230 mW m^-2^ nm^-1^ at 370–380 nm (UV) using a High-Speed Polychromator System VisiChrome (Visitron System GmbH, Puchheim, Germany) which allows illumination with monochromatic light. To simulate higher intensity “daylight” we used a KL1500LCD lamp, which reaches intensities at place of 100–300 (μmol photons m^-2^ s^-1^) with a broad light spectrum (data available from Schott AG, Mainz).

In 72 experiments, oxygen evolution and utilization in cultures was measured simultaneously, in the dark, with and without the addition of 30 μmolL^-1^ Ageladine A and after a 90 minute exposure to UV light. Measurements were conducted using subsamples from the same aliquot to run the experiments in parallel. The latter allows direct comparison with modelling results. In preliminary tests, an incubation with 30 μmolL^-1^ Ageladine A for at least two hours prior to the experiments gave the best results. These concentrations are in the range found in naturally occurring sponges where 5 mg Ageladine A was previously isolated from one Agelas sponge.

Experiments were performed at room temperature (22 ± 1.5°C) Ageladine A used was synthetized by Thorsten Koschmieder (nee Mordhorst) at the Alfred Wegener Institute in Bremerhaven [11]. Preliminary tests revealed no influence of the fluorescent dye Ageladine A on the response of the oxygen optodes. DMSO (<0.5%) was used as Ageladine A solvent and was always used in controls (vehicle control). Differences between simultaneous treatments were assessed using a students t-test and *p*<0.05 was considered significant. The following definitions are used: O_2_ respiration rate consisted of the alteration of O_2_ in percent per hour (of 100% air saturation). Gross O_2_ production rate: alteration of O_2_ in percent per hour derived from photosynthesis. Net O_2_ production rate: alteration of O_2_ in percent per hour derived from photosynthesis and respiration. O_2_% saturation: starting with 100% air saturation a measure of respiration and O_2_ production.

### Electron transport-irradiance curve to check for impacts of Ageladine A on PSII

Treatment effects on the photophysiology of *S*. *bacillaris* were assessed with a fast repetition rate fluorometer (FRRf) in combination with a FastAct Laboratory system (FastOcean PTX), both from Chelsea Technologies Group ltd. (West Molesey, UK). The measurements were taken at 20° C in a temperature controlled room following a 10 min dark acclimation period, assuring that all photosystem II (PSII) reaction centers were fully oxidized and nonphotochemical quenching was relaxed [19]. Excitation wavelength of the fluorometer’s LED was 450 nm with an automated adjustment of the light intensity to 0.66–1.2 x 10^22^ μmol photons m^-2^ s^-1^. A single turnover mode with 100 flashlets saturation phase on a 2 μs pitch and 40 flashlets relaxation phase on a 40 μs pitch was used to increasingly saturate PSII. Iterative algorithms for the induction [20] and relaxation phases [21] were applied to estimate minimum Chl a fluorescence (F_0_) and maximum Chl a fluorescence (F_m_). The apparent maximum quantum yield of photosynthesis of PSII (F_v_/F_m_) could then be calculated according to the equation

$$\frac{{\text{F}}_{\text{v}}}{{\text{F}}_{\text{m}}}=\left(\frac{{\text{F}}_{\text{m}}-{\text{F}}_{0}}{{\text{F}}_{\text{m}}}\right)$$ (1)

Photosynthesis-irradiance-curve (PE-curves) were generated using 8 levels of irradiances between 0 and 1038 μmol photons m^-2^ s^-1^ with a 5 min acclimation to each light level followed by six subsequent Chl a fluorescence measurements at each light level. From these measurements, the light-adapted minimum (F′) and maximum (F_m_′) fluorescence of the single turnover acquisition was estimated. The effective PSII quantum yield under ambient light (F_q_'/F_m_') was derived according to the equation (F_m_′ − F′) / F_m_' [22]. Absolute electron transport rates (aETR, e^-^ PSII^-1^ s^-1^) for each light level were calculated according to [23] and [24] from σ_PSII_ x [(F_q_'/F_m_′)/(F_v_/F_m_)] x I, where σ_PSII_ is the functional absorption cross section of PSII’s photochemistry (nm^2^ quanta^-1^) and I denotes the instantaneous irradiance (photons m^-2^ s^-1^). The aETRs vs. irradiances were then curve fitted according to [25], which takes into account possible photoinhibition, and photosynthetic parameters, including minimum saturating irradiance (Ik), the potential maximum aETR (aETR_max_), and the maximum light utilization efficiency (α).

### Confocal imaging

A Leica SP5 equipped with several lasers including a multiphoton laser was used for obtaining images. Tissue samples from *Agelas wiedenmayeri* were collected from the Caribbean, dried and frozen at -80° C. They were gently thawed in seawater and the transferred to the microscope. A 405 nm diode laser was used to acquire the images while a wavelength scan mode was used to measure the emission wavelength profile. A projection of a z-stack of 228 μM (z) and 1,55 X 1,55 mm (X, Y) was used.

## Results

In order to calculate and model the effects of Ageadine A, its quantum yield was measured. The quantum yield of Ageladine A was calculated using Quinine Sulfat Dihydrate in 0.5 mol H_2_SO_4_, as standard, with a known quantum yield of 0.546 using the value and formula of [26]:

$$\varphi_{f}^{i}=\frac{F^{i}f_{s}n_{i}^{2}}{F^{s}f_{i}n_{s}^{2}}\varphi_{f}^{s},$$ (2)

where *φ*^*i*^_*f*_ and *φ*^*s*^_*f*_ represent the Quantum yield of probe and standard. *F*^*i*^ and *F*^*s*^ represent the integral of the emitted fluorescence spectra of the probe and standards, whereas *f*_*i*_ und *f*_*s*_ are factors of the absorption spectra. *fx* is the fraction of the light impinging on the sample that is absorbed (*fx* = 1 − 10^–*Ax*^, where *A* = absorbance) while *n*_*i*_ and *n*_*s*_ represent refraction indices of the solvent [26]. The standard as well as Ageladine A were excited at 366 nm, resulting in a *φ*^*i*^_*f*_ of 0,87 in artificial sea water with a pH of 4 [27].

Because no isolate of sponge derived *Synechococcus* is available, this study presents a proof of concept using a readily available strain of *Synechococcus bacillaris* (Butcher, CCMP1333). Cultures were exposed to UV light (370-380nm) and oxygen production, as a proxy for photosynthesis, was assessed with and without the addition of Ageladine A (30 μmolL^-1^). 90 minutes of exposure to UV light resulted in a highly significant (p<0.002, n = 20) increase of gross O_2_ production in the Ageladine A treatments vs. the controls (2.34 ± 0.38 and 0.92 ± 0.32% h^-1^, respectively; Fig 1b). During the initial 10h dark acclimation period, no significant difference in O_2_ respiration rates between the controls and the treatments was observed (1.61 ± 0.11 and 1.56 ± 0.15% h^-1^, respectively; Fig 1c), excluding an effect of Ageladine A on respiration. 20 experiments with the highest respiration rates (≥1% h^-1^) measured during a preceding overnight dark acclimation period were chosen and presented (Fig 1b).

**Fig 2:**
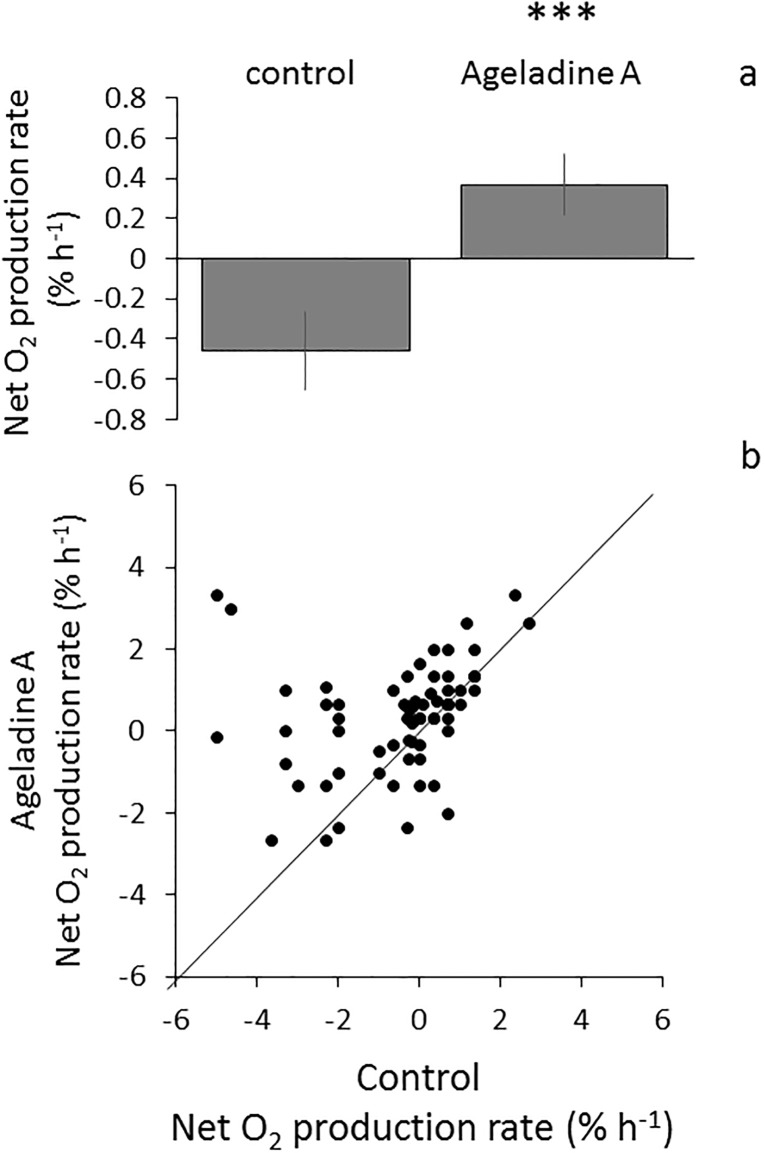
a. All experimental data w/o overnight treatment and differing incubation time. Net O_2_ production rate of medium during UV exposure of Ageladine A treated cells and controls (N = 72). Paired t test P value 0,0002 *** Two-tailed. b: The same data as in a: The control value represent the X-axis of one experiment, the Ageladine A treatment of the same experiment is on the Y—axes. At especially low control values (X-axis) the treatment (Ageladine A) values are comparably high (Y-axis).

Combining all 72 experiments yielded a significant (p = 0.0002) increase of O_2_ production with UV exposure in the Ageladine A vs. the controls. This was the case even when data without overnight incubation and short term incubation >2h were pooled. Fig 2a and 2b shows data of all 72 experiments with net O_2_ production rates of the control and Ageladine A treatment (control -0.456 ± 1.634; Ageladine 0.369 ± 1.29% h^-1^. Analysis revealed that in experiments where the O_2_ content after 90 minutes of light exposure was lowest, the addition of Ageladine A showed the largest effect (Fig 2b).

**Fig 3:**
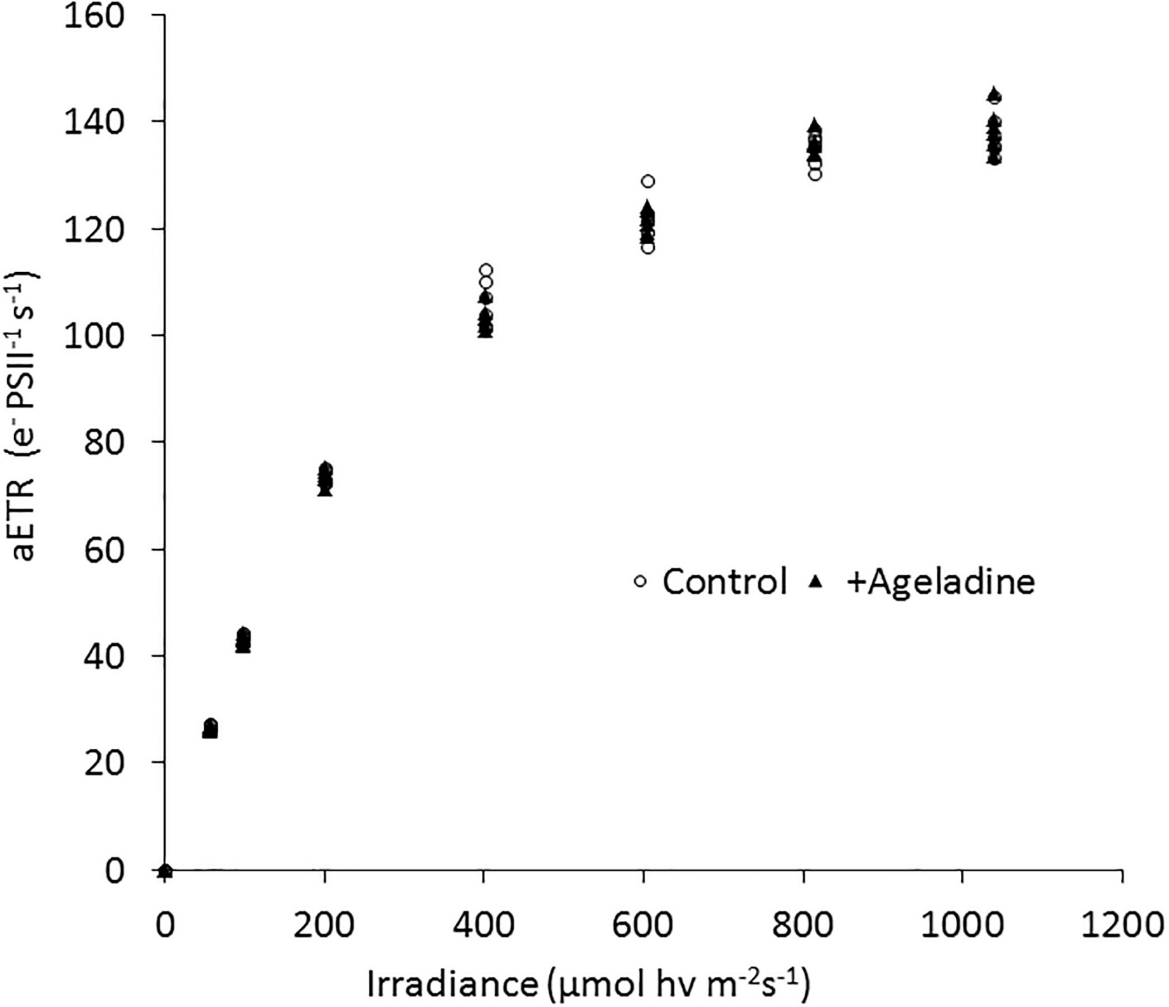
Effects of the addition of 30 μM Ageladine A on the absolute electron transport rates (aETR, e- PSII^-1^ s^-1^; a, b) of *Synchecoccus bacillaris* in response to increasing instantaneous irradiance, measured using a fast repetition rate fluorometer (FRRf). No measurable effect of the addition of Ageladine A on the aETR was observed.

In order to assure that neither the addition of Ageladine A nor the exposure to UV light impacted the photophysiology of *Synchecoccus*, a photosynthesis vs. irradiance (PI) curve was measured for cultures with and without the presence of Ageladine A using a fast repetition rate fluorometer (FRRf). The latter highlighted that the electron transport rate of photosystem II (PSII) under normal irradiance was not affected by the presence of Ageladine A (Fig 3).

**Fig 4:**
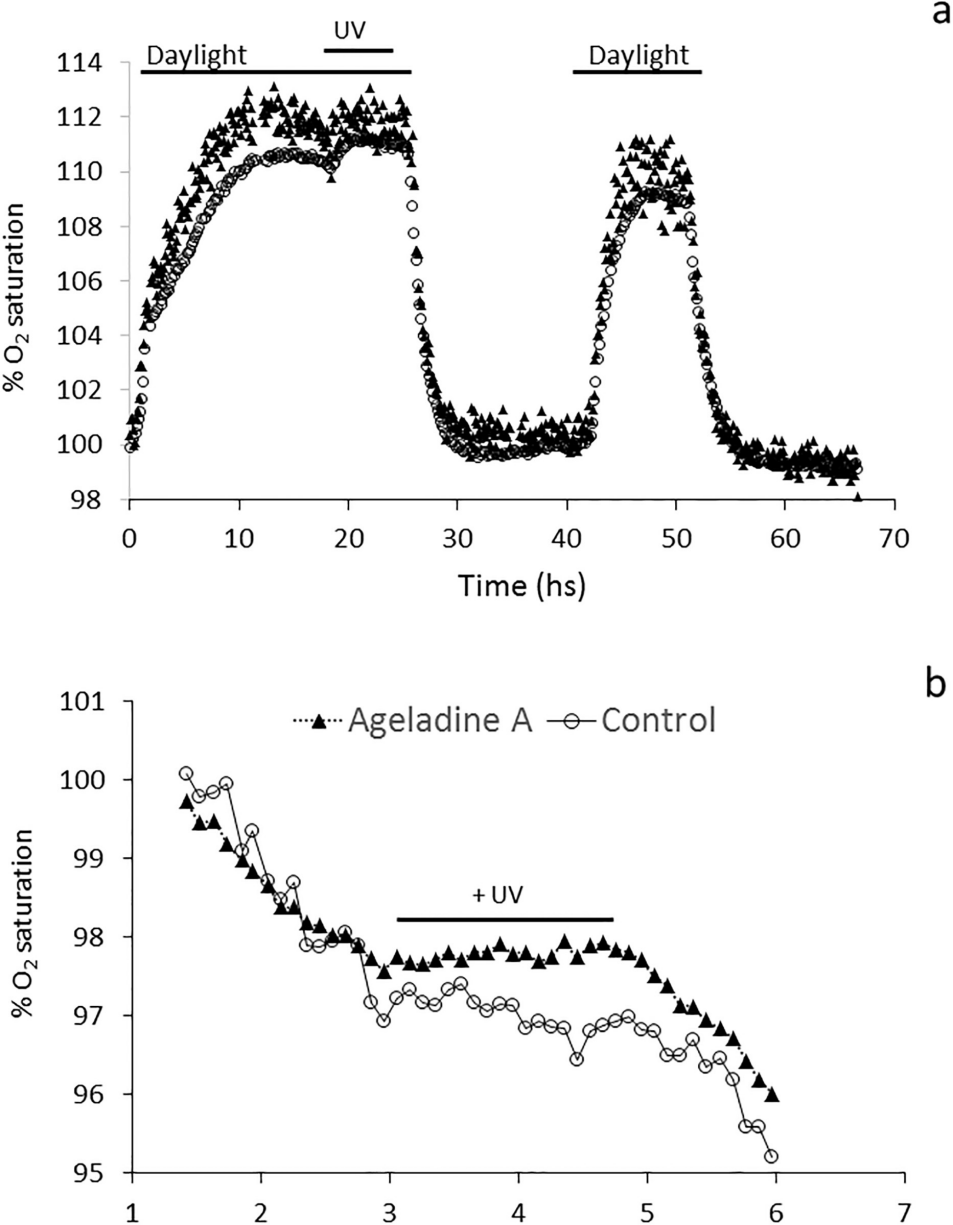
The impacts of Ageladine A and exposure to UV light on the gross oxygen production of *Synechococcus bacillaris*. **a**: % oxygen saturation before and after UV exposure indicating a lack of photo damage. **b**: % change in oxygen saturation over time for treatments with and without the addition of Ageladine A; cuvettes were subjected for 90 mins to 30 μmol photons m^-2^ s^-1^ of UV radiation (370–380 nm).

This was also supported by the fact that at the end of the experiment UV treated cells, exposed to “daylight” (broad spectrum, 100–300 (μM hv m^-2^ s^-1^), yielded a similar net O_2_ production, irrespective of the presence of Ageladine A (Fig 4a). Together with our FRRf measurements, we concluded that neither photo damage due to UV nor the Ageladine itself played a factor. The change in oxygen saturation over time for treatments with and without the addition of Ageladine A is shown in Fig 4b.

The impact of Ageladine A on photosynthetic electron transport rates (ETRs) and thus oxygen evolution in *Synechococcus* was mathematically modeled and compared to the experimental results. An existing model of photosynthetic electron transport rates (ETR) by [28] was modified by including Ageladine A in the description of the antennae system. The main accessory pigments of cyanobacteria are phycobilisomes, which absorb light at longer wavelengths. Of these, the predominant phycobiline in *Synechococcus* is phycocyanin, which absorbs light at 618 nm. Because of the negligible spectral overlap of Ageladine A fluorescence and phycocyanin absorption, phycobilisome pigments were not taken into account for this model. The number of chlorophyll a (Chl a) molecules in PSII (N_chla,II_) and PSI (N_chla,I_) are ~40 PSII^-1^ and ~100 PSI^-1^, respectively [29]. The number of Ageladine A molecules per PSII and PSI was estimated from the accumulation of Ageladine A inside the acidic thylakoid lumen. In seawater (pH = 8.1), the major Ageladine A species is the neutral form (~93%) [12]. Because of its membrane permeability, the neutral form enters the thylakoid lumen until equilibrium is reached. At the acidic lumenal pH = 4.5 [14], the singly and doubly charged Ageladine A species are dominant (~97%), whereas the neutral form is present only in small concentrations (0.01%). In this study, the total Ageladine A concentration of 30 μmolL^-1^ in the medium, thus resulted in an accumulation of 0.27 molL^-1^ fluorescing Ageladine A species inside the thylakoid lumen. Adjacent to the lumen, PSII and PSI cores consist of 8 nm diameter disks [30]. If the lumen has a thickness of 10 nm and it is assumed that half of the thickness serves each core, then the number of fluorescent Ageladine A molecules in the 50.27 × 5 nm^3^ lumen elements adjacent to PSII (N_Agel,II_) and PSI (N_Agel,I_) is 41 PS^-1^.

At spectral regions, where the absorption by Chl a is high, (~ 440 nm), the optical cross section of Chl a (σ_chla_) is 8.67× 10^−21^ m^2^ Chl a^-1^ [31]. In comparison, at the 380 nm used in this study, absorption was reduced by a factor of 2.2–2.5 [32, 33]. Using the conservative value of 2.2, σ_chla_ at 380 nm was 3.94×10^−21^ m^2^ Chl a^-1^. By means of light absorption measurements, it was possible to determine the optical cross section of Ageladine A (σ_Agel_). The measured absorbance of 30 μmolL^-1^ Ageladine A (pH = 4) (c) contained in a 1 cm (d) thick cuvette (A, absorbance) was 0.307. Using Beer-Lambert’s law of light absorption, σ_Agel_ was calculated as follows:

$$\sigma_{\text{Agel}}=\frac{\text{A}}{\text{cd}}\times \frac{\text{ln}\left(10\right)}{{\text{N}}_{\text{A}}}=3.91\times 10^{-21}{\text{m}}^{2}{\text{Agel}}^{-1},$$ (3)

where N_A_ = 6.022 × 10^23^ mol^-1^ is Avogadro’s constant. The optical cross sections for PSII (σ_PSII_) and PSI (σ_PSI_) are given by:

$$\sigma_{\text{PSII}}=\sigma_{\text{chla}}{\text{N}}_{\text{chla},\text{II}}+\gamma \sigma_{\text{Agel}}{\text{N}}_{\text{Agel},\text{II}},$$

$$\sigma_{\text{PSI}}=\sigma_{\text{chla}}{\text{N}}_{\text{chla},\text{I}}+\gamma \sigma_{\text{Agel}}{\text{N}}_{\text{Agel},\text{I}},$$ (4)

where *γ* = 0.87 is the quantum yield of the Ageladine A fluorescence.

**Fig 5:**
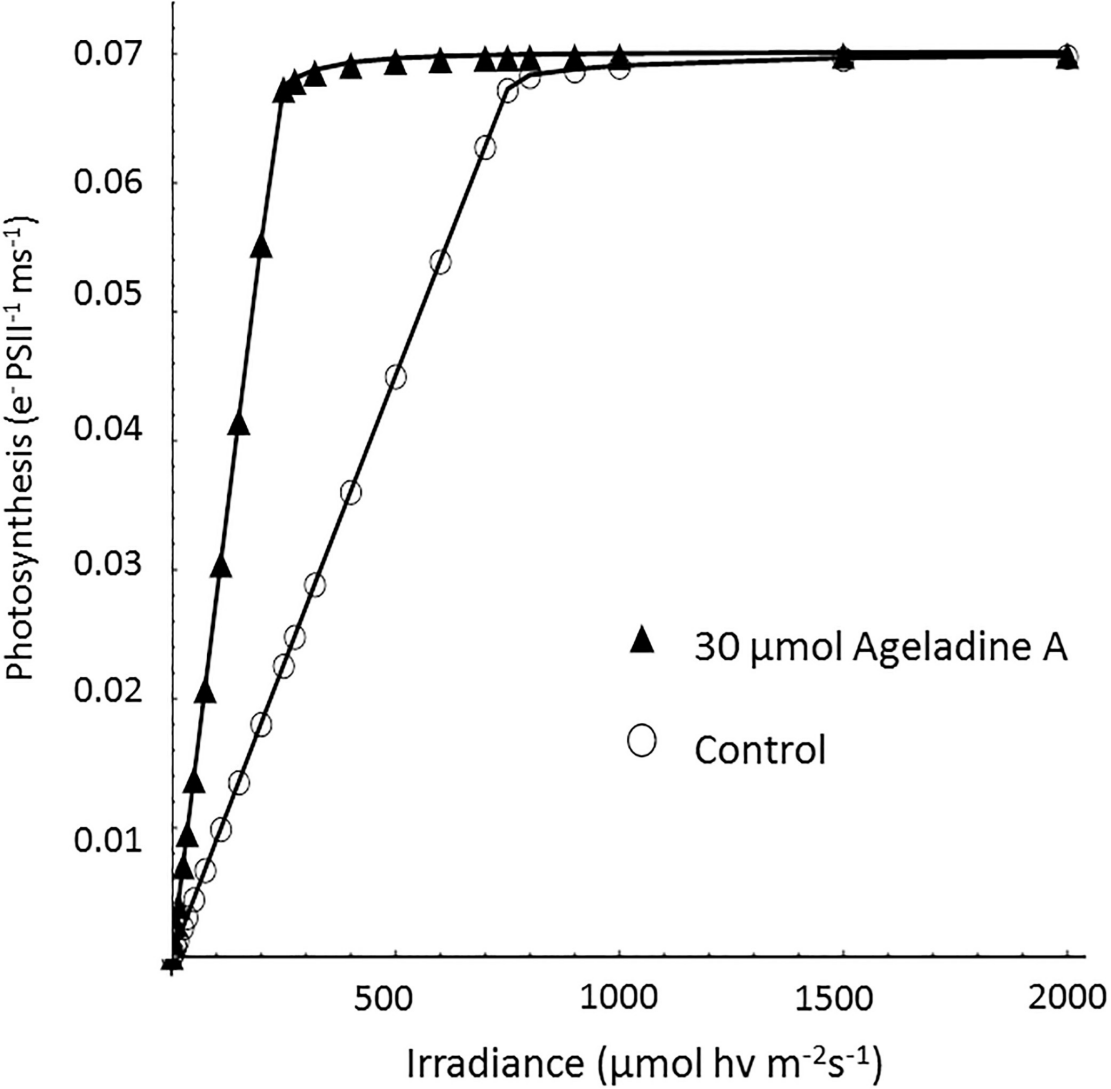
Theoretical simulation of photosynthesis as a function of irradiance based on the model of [28]. The number of Chl a molecules in PSII and PSI were 40 and 100, respectively [29]. The number of Ageladine A molecules in both PSII and PSI were modelled based on the accumulation of Ageladine A inside the acidic thylakoid lumen. For the antenna scenario without Ageladine A (open circles) the cross sections for PSII (σ_PSII_) and PSI (σ_PSI_) are 1.58 10^−19^ m^2^ and 3.94 10^−19^ m^2^, respectively. With Ageladine A (closed triangles) σ_PSII_ and σ_PSI_ are 4.86 10^−19^ m^2^ and 1.01 10^−18^ m^2^, respectively. The value for the rate constant of ferredoxin re-oxidation was 0.12 ms^-1^ [28] for both antenna scenarios.

The ETR, as a function of photon flux (PFD, in μmol photons m^-2^ s^-1^), was calculated with the help of the photosynthesis model of [28]. The theoretical curves of the ETRs with and without Ageladine A are shown in Fig 5. While ETRs remained unaffected by Ageladine A at very high PFDs, under limiting light conditions, the ETRs were substantially increased due to Ageladine A. These results suggest that Ageladine A can help increase the rate of photochemistry of the symbionts under low light conditions. The model calculations are compared to the experimental results obtained for irradiance values in units of W m^-2^. The conversion from irradiance to PFD given in mol photons m^-2^ s^-1^ is

$$I^{E}\left(\lambda \right)=I^{W}\left(\lambda \right)\times \frac{\lambda }{N_{A}hc},$$ (5)

where *I*^*E*^(*λ*) and *I*^*W*^(*λ*) are the spectral photon flux and spectral irradiance, respectively, *λ* is the wavelength of the incident light, N_A_ = 6.022 × 10^23^ mol^-1^ is Avogadro’s constant, h = 6.626 × 10^−34^ Js is Planck’s constant, and c = 2.998 × 10^8^ms^-1^ is the speed of light. The Superscript ‘E’ refers to Einstein (E = mol photons), a unit often used in the photophysiological community. Then, the PFD is calculated by integrating *I*^*E*^(*λ*) over all wavelengths. O_2_ evolution was detected in cultures of *Synechococcus* at *I*^*W*^(*λ* = 380 nm) = 230 mW m^-2^ nm^-1^. The corresponding PFD was *I*^*E*^(*λ* = 380 nm) × Δ*λ* = 26 *μ*mol photons m^-2^s^-1^, with Δ*λ* = 36 nm the spectral line width of the light source. For PFD = 26 *μ*mol photons m^-2^s^-1^ the model results (Fig 5) indicate an Ageladine A induced increase of ETR, and thus O_2_ production, by a factor of 3.1, which is comparable with the experimental value of 2.54. It is important to note that emission from molecules in solution is usually isotropic, so some significant fraction of the emission may be directed away from the core. The later may be one reason for the discrepancy between the measured and calculated factor.

## Discussion

The advantages for sponges having photosynthetic symbionts are well known, mainly that the symbionts provide the sponge with metabolites and carbohydrates, increasing their food supply [34]. Additionally in warm and hypoxic waters, O_2_ production by *Synechococcus* might also be advantageous for sponges. Sponges, in turn, provide inorganic nitrogen and organic carbon to their symbionts, which may be especially critical in oligotrophic waters [35]. While Faulkner et al. [36] suggests, that few secondary metabolites, are the result of host/symbiont interaction, Brinkmann et al. [37] argues that “most sponge-derived bioactive compound syntheses are the result of a cooperative efforts between both the sponge host and their microbial symbionts, and their synthesis may thus be triggered by the precursor compounds supplied by either the host or the symbiont.” Even though the origin of Ageladine A is still unknown, it is likely a function of the symbiotic relationship between the sponge and its microbiome.

This study suggests that light may also serve as a symbiotic currency. *Agelas* sponges have a global distribution, growing at different depth. Life at different depths exposes its symbionts to possible light limitation, since major components of PAR are absorbed at relatively shallow depths. In algae and higher plants, accessory pigments fill the ‘green gap’, the region between the two Chl a absorption maxima (430 and 630 nm), thereby optimizing access to light [38]. Ageladine A may serve a similar function, converting a previously unusable and potentially damaging part of the light spectrum (UV) into usable PAR. The model highlights that Ageladine A can help increase the photosynthetic rate of the symbionts under suboptimal light (Fig 5). Since the photosynthetic ETRs are directly proportional to the rates of O_2_ production by the symbionts, Ageladine A could possibly promote ATP synthesis by the sponge via increased respiration of the sponge. This would be especially true if O_2_ is limiting, i.e. in warm, hypoxic waters.

**Fig 6:**
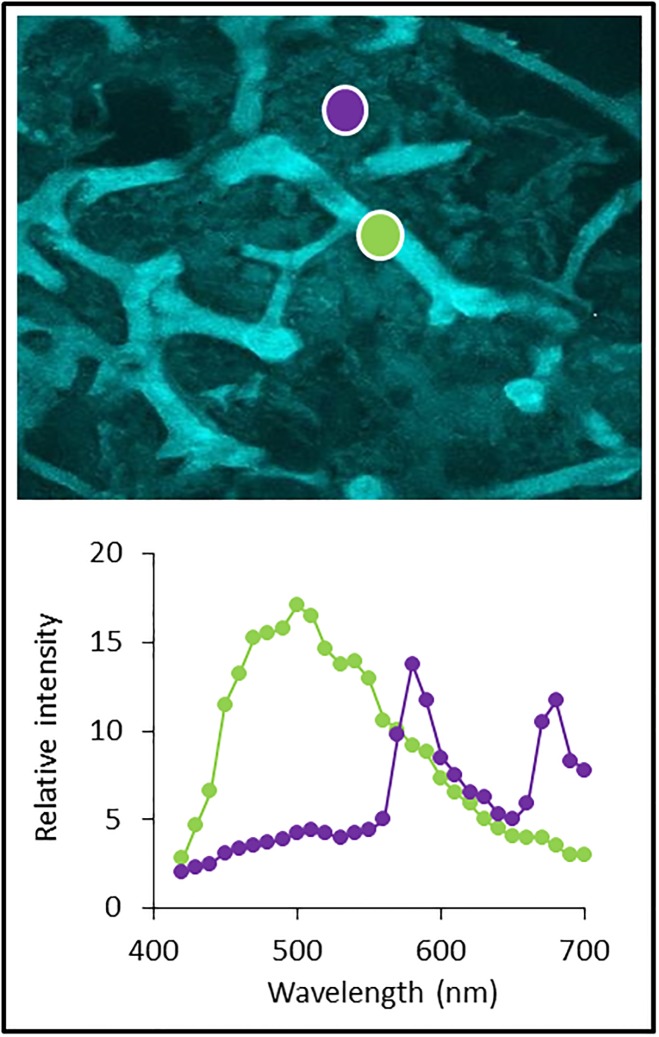
Spongin fibres and tissue of a *Agelas wiedenmayeri* sponge visualized with a confocal microscope (excitation at 405 nm). The violet and the green spots indicate the region of interest where an emission wavelength scan was performed. The spongin fibres fluoresce strongest at 500 nm, whereas fluorescence in between is around 580 nm and 680 nm, suggesting the presence of phycoerythrin and chlorophyll.

Croce and Amerongen [38] commented in *Natural strategies for photosynthetic light harvesting* that ‘evolution has led to many different solutions in very diverse environments but has done so by combining a relative small number of building principles’. Similarly to our study, they describe a mosaic approach, combining light harvesting molecules from different sources, to build a system, which may best fit a specific environmental niche. Furthermore, Ageladine A is not the only fluorescing compound found in sponges [39,40]. The spongin fibres themselves fluoresce, not only in the blue/green range (Fig 6; this work) but also in the red [41], potentially exciting Chl a as well as other accessory pigments. Agelas sponges belong to the demospongae, who’s primary structural components consist of spongin. This compound also fluoresces in the blue to green range when excited with 405 nm light (Fig 6), and, like the fiber optical properties of a glass sponge spicule [42], spongin may facilitate light transmission into the interior of the sponge. Our spongin fluorescence measurements were performed using a diode laser whose light intensity is not comparable to in situ light conditions. Thus while our results are not quantitative, they nevertheless support the idea that these processes potentially support photosynthesis by *Synechococcus* and/or other symbionts. Gao et al. 2014 wrote: “These genetic modifications imply that “*Ca*. *Synechococcus spongiarum*” SH4 represents a low-light adapted cyanobacterial symbiont and has undergone genome streamlining to adapt to the sponge’s mild intercellular environment.” Therefore it seems possible, that the secondary metabolite Ageladine A may have overtaken functions in the species to effectively perform photosynthesis in a light limited environment. Additionally Ageladine A may be an UV protective for low light adapted symbionts and additionally support photosynthesis [43]. Most common UV protectors in marine organisms are Mycosporine-like amino acids (MAAs), which do not fluoresce and absorb between 285–360 nm, possibly interfering with Ageladine A function [44]. However the enhancement of photosynthesis is probably not altered significantly since the absorption maximum of Ageladine A is at 370-380nm.

All of our experiments were performed with *Synechococcus* strain WH5701 (CCMP1333), which may well be inferior to sponge *Synechococcus* evolved in symbiosis with sponges. Even a few percentage of additional light energy yield may be a major advantage for Agelas sponges against competing organisms. Nevertheless, light spectral measurements in the sponge tissue are required to validate that there is sufficient UVA in vivo to significantly enhance photosynthesis.

Photosynthesis in microalgae has likely undergone multiple evolutionary twists and turns and the idea of light energy being harvested by a protist and being passed on to its animal host is by no means novel. Although we do not know if the sponge alone or a complex microbial interaction is responsible for the production of Ageladine A, the interesting aspect of this study is the prospect of an animal producing compounds which alter the light in such a way as to maximize photosynthetic products by its symbiont inhabitants. Thus while the majority of studies investigating symbiosis focus on the exchange of chemical compounds, this study suggests a form of symbiosis, based on light. In situ investigations should follow this pilot study to validate our hypothesis under natural conditions.
